# Attentional Control and Intelligence: MRI Orbital Frontal Gray Matter and Neuropsychological Correlates

**DOI:** 10.1155/2015/354186

**Published:** 2015-05-26

**Authors:** Paul G. Nestor, Motoaki Nakamura, Margaret Niznikiewicz, James J. Levitt, Dominick T. Newell, Martha E. Shenton, Robert W. McCarley

**Affiliations:** ^1^Department of Psychology, University of Massachusetts, Boston, MA 02401, USA; ^2^Clinical Neuroscience Division, Laboratory of Neuroscience, Boston VA Healthcare System-Brockton Division, Department of Psychiatry, Harvard Medical School, Brockton, MA, USA; ^3^Psychiatry Neuroimaging Laboratory, Department of Psychiatry, Brigham and Women's Hospital, Harvard Medical School, Boston, MA, USA

## Abstract

Attentional control is a key function of working memory that is hypothesized to play an important role in psychometric intelligence. To test the neuropsychological underpinnings of this hypothesis, we examined full-scale IQ, as measured by the Wechsler Adult Intelligence Scale-Third Edition (WAIS-III), and attentional control, as measured by Trails B response time and Wisconsin Card Sorting (WCS) test perseverative errors in 78 healthy participants, 25 of whom also had available magnetic resonance imaging (MRI) gray matter volume studies of the orbital frontal cortex (OFC) parcellated into three regions: gyrus rectus, middle orbital gyrus, and lateral orbital gyrus. Hierarchical regression indicated that Trails B response time specifically explained 15.13% to 19.18% of the variation in IQ and WCS perseverative errors accounted for an additional 8.12% to 11.29% of the variance. Full-scale IQ correlated very strongly with right middle orbital gyrus gray matter volume (*r* = 0.610, *p* = 0.002), as did Trails B response time with left middle orbital gyrus gray matter volume (*r* = −0.608, *p* = 0.003). Trails B response time and right middle orbital gyrus gray matter volume jointly accounted for approximately 32.95% to 54.82% of the variance in IQ scores. These results provided evidence of the unique contributions of attentional control and OFC gray matter to intelligence.

## 1. Introduction

Intelligence is a well-established predictor of important life outcomes ranging from school performance, occupational status to adult health and longevity (e.g., [1, 2]). Its psychometric measurement in the form of IQ tests is perhaps the most reliable index of individual differences in psychology [3, 4], although its underlying neurological organization has yet to be fully elucidated. Over the past two decades, however, there has been considerable progress in brain imaging and cognitive neuroscience approaches directed towards the neuropsychological study of individual differences in IQ. These studies have begun to elucidate some of the critical neurodevelopmental (e.g., [5]), neuroanatomical (e.g., [6]), and cognitive (e.g., [7]) mechanisms underlying variation in IQ test scores.

In particular, from a cognitive perspective, working memory has been consistently linked to intelligence, estimated to account for about 50% of the variance in IQ test scores [7]. Studies have shown that this relationship may, in turn, be mediated by a rather specific set of working memory processes related to executive attentional control that allow for stimulus representations to be actively maintained on-line in the context of distraction and interference (e.g., [8, 9]). In fact, Kane et al. [10] proposed attention-control capacity as the “secret ingredient” that is recruited by working memory tasks and largely explains the relationship of working memory and intelligence (see also [11]).

As a central component of working memory, attentional control is conceptualized and defined as part of an executive system for organizing and planning goal-directed behavior and intellect [12]. Findings from structural and functional brain imaging studies have suggested that intelligence as well as attentional control processes of working memory each depends heavily on neural circuitry of the prefrontal lobe [13]. For intelligence, the prefrontal cortex is seen as a key hub in a widely distributed network of brain areas spanning temporal and parietal sites that supports high-order cognition [14]. In a similar vein, findings from functional imaging studies have provided evidence that attention-control capacity may be decomposed into regulative and evaluative components, each supported by distinct regions within the prefrontal cortex. That is, a regulative component, recruited to coordinate the demands of activation, inhibition, and switching, relies heavily on orbital frontal and lateral prefrontal subdivisions, whereas medial frontal sectors are recruited for monitoring and signaling adjustments in control [15]. However, the precise contributions of these attentional control processes and their neural circuitry to intelligence have yet to be fully established.

The current study thus aimed to examine individual differences in psychometric intelligence in relation to attentional control and its underlying prefrontal sources. We employed a multimodal research design that combined neuropsychological measures of intelligence and attentional control with structural magnetic resonance imaging (MRI) of prefrontal lobe regions. The Wechsler Adult Intelligence Scale-Third Edition (WAIS-III) full-scale IQ provided a measure of general intelligence, and Trails B of the Trail Making Test (TMT) and perseverative errors on the Wisconsin Card Sorting (WCS) test served as indices of attentional control. Trails B, a speeded, paper-and-pencil task, which involves connecting alternating numbered circles and lettered circles, places heavy demands on attentional control processes related to response inhibition, task switching, and shifting mental set [16]. Similarly, WCS perseverative errors are presumed to reflect a failure to inhibit or override a previously correct sorting rule in the face of real-time performance feedback [17]. Importantly, from a construct validity perspective, Trails B and WCS represent two different forms of measurement of the same construct, attentional control. That is, Trails B uses a timed pencil-and-paper format and the WCS test uses an untimed, multiple-choice format. For construct validity, the aim is to use different measures of the same construct so that unwanted method variance can be minimized while hypothesized trait variance, which in this study is defined as attentional control, can be maximized, and its role in intelligence can be quantified.

How might individual differences in attentional control and intelligence be related to normal structural variation in key prefrontal lobe sites? Among the most polymodal regions of the brain in general, and the prefrontal lobe in particular, is the orbital frontal cortex (OFC) located between the frontopolar gyri rostrally, the anterior perforated substance caudally, the inferior frontal gyrus laterally, and the ventromedial margin of the cerebral hemisphere medially [18, 19]. The OFC receives multisensory inputs of taste, smell, auditory, visual, and somatosensory as well as visceral signals, due to its wide and deep connections to functionally diverse cortical and subcortical regions, including the amygdala, cingulate cortex, insula, hypothalamus, hippocampus, and striatum, as well as its neighboring dorsolateral prefrontal cortex [20]. Its anatomically heterogeneous sulcogyral morphology [19, 21, 22] is thought to be reflective of the rich molecular processes underlying neuronal migration, local neuronal connection, and synaptic development, as well as lamination and formation of cytoarchitecture [23, 24].

In the present study, we use a within-subjects design that combines neuropsychology and MRI structural measures to test the attentional control hypothesis of intelligence. To do so, we first examine in a large sample of healthy participants the relationship of intelligence to performance on two indexes of attention-control processes that are important for response inhibition and shifting mental sets—Trails B and WCS perseverative errors. The pivotal research question addressed here is the extent to which individual differences in intelligence can be independently and specifically accounted for by performance on these measures of attention-control capacity. Second, OFC gray matter volumes are examined in relation to both attentional control process and intelligence in a smaller subset of these healthy controls from our prior studies [25, 26] who had available structural MRI studies. Here we aimed to quantify the relative and unique contributions of OFC brain structures and attention control processes to intelligence.

## 2. Method

All participants were between the ages of 21 and 58 years, right-handed, native speakers of English, without histories of electroconvulsive therapy and neurological illness, and without alcohol or drug abuse in the past 5 years. Recruited as healthy comparison subjects for prior neuropsychological studies of veterans with schizophrenia (e.g., [26]), all participants (*N* = 143) met Structured Clinical Interview for DSM-IV Axis I Disorders-Non-patient Edition (SCID-NP) criteria of no past or current Axis 1 and/or Axis II disorder [27, 28]. Participants had a mean age of 40.83 years (S.D. = 9.06) and a mean education of 14.89 years (S.D. = 2.06). All participants gave informed consent. The neuropsychological tests were administered at the Boston VA Medical Center (Brockton, MA Division) and the MRI studies were conducted on a subset of participants (*N* = 25) at the Brigham and Women's Hospital in Boston, MA. MRI studies and neuropsychological testing were completed over the course of approximately three months. The research protocol was approved by the Institutional Review Board of the Boston VA Medical Center and Harvard Medical School.

The neuropsychological battery included (1) Trail Making Test (TMT; [16]); (2) Wisconsin Card Sorting Test (WCS; [17]); and (3) Wechsler Adult Intelligence Scale-Third Edition (WAIS-III; [29]). The study used a within-subjects design, meaning that the same subjects completed the three neuropsychological tests. That is, 138 participants completed both the TMT and the WCS, 94 of these same participants also completed the TMT and WAIS-III, 81 completed the WCS and the WAIS-III, and 78 completed all three measures.

### 2.1. MRI Processing

MRI studies were available for 25 healthy right-handed, participants (19 males/6 females) who served as normal comparison subjects for prior MRI studies of veterans with schizophrenia (e.g., [25]) and these same MRI data have been used in other studies of healthy cognition [26]. The MRI processing is described in detail in Nakamura et al. [25]. In brief, MR images were also acquired with a 1.5-Tesla General Electric scanner (GE Medical Systems, Milwaukee) at the Brigham and Women's Hospital in Boston. A three-dimensional Fourier transformed spoiled gradient-recalled (SPGR) acquisition sequence yielded a coronal series of contiguous 1.5 mm images (TE = 5 msec, TR = 35 msec, repetition = 1, nutation angle = 45°, field of view = 24 cm, acquisition matrix = 256 × 256 × 124, and voxel dimension = 0.9375 × 0.9375 × 1.5 mm). Next, a double-echo spin-echo yielded 108 contiguous axial double-echo (proton-density- and T2-weighted) slices, with 54 levels, throughout the brain (TE = 30 and 80 msec, TR = 3000 msec, field of view = 24 cm, an interleaved acquisition with 3-mm slice thickness, and voxel dimensions = 0.9375 × 0.9375 × 3.0 mm). The T2 information from the double-echo spin-echo axial slices was registered to the SPGR images. An expectation-maximization (EM) segmentation technique [30] was used to segment the images into three major tissue classes: gray matter; white matter; and CSF, using both SPGR and T2-weighted MR information as well as spatial priors. This technique was used to extract Intracranial Contents (ICC) volume. Manual tracing of OFC ROI was performed on nonsegmented images to avoid segmentation errors due to susceptibility artifacts which are common in the OFC region.

Images were realigned using the line between the anterior and posterior commissures and the sagittal sulcus to correct head tilt and resampled into isotropic voxels (0.9375 mm^3^). This realignment procedure was essential for precise and consistent ROI delineation. Three-dimensional information was used to provide reliable delineation of the OFC ROI with a software package for medical image analysis [3D slicer, http://www.slicer.org] on a workstation. Definition and details of the method of region of interest for the OFC are provided in Nakamura et al. [25]. In brief, the OFC is anatomically heterogeneous in the enormous interindividual variability that characterizes its sulcogyral morphology [19, 21, 22]. Given such remarkable interindividual structural variation, we in a prior MRI study in schizophrenia used two of the most stable and reliably imaged sulci, the olfactory sulcus and the lateral orbital sulcus, as boundaries to divide the OFC into three subregions: gyrus rectus, middle orbital gyrus, and lateral orbital gyrus [25]. All manual delineations were performed by Nakamura et al. Intraclass interrater reliability correlation coefficients based on seven randomly chosen cases were 0.95 for left gyrus rectus and 0.96 for right gyrus rectus; 0.99 for left middle orbital gyrus and 0.96 for right middle orbital gyrus; and 0.96 for left lateral orbital gyrus and 0.99 for right lateral orbital gyrus.

### 2.2. Statistical Analysis

Pearson's correlations evaluate the univariate relationships between neuropsychological measures as well as those between neuropsychological measures and MRI gray matter volumes. Hierarchical regression analyses examine the joint and unique influences of specific neuropsychological measures of attentional control (i.e., Trails B response time, WCS perseverative errors) on intelligence, as measured by the WAIS-III full-scale IQ. We then use hierarchical regression to test the attentional control hypothesis of intelligence entering independent variable of OFC gray matter volume and Trails B with WAIS-III full-scale IQ as the dependent variable. Hierarchical regression analyses computed partial (*r*p) and semipartial (*r*sp) correlations which allow for partitioning total variance of the dependent variable of full-scale IQ among the independent variables of OFC gray matter volume and Trails B response time. The partial correlation squared (*r*p^2^) and semipartial correlation squared (*r*sp^2^) quantify the proportion of variance in full-scale IQ that is uniquely and specifically explained by each of the independent variables, OFC gray matter volume and Trails B response time. In conjunction with other linear regression statistics, partial and semipartial correlations provide a comprehensive picture of how OFC gray matter volume and Trails B response time relate to full-scale IQ when collinearity is controlled. For all regression analyses, the *F*-to-enter probability was 0.05 and the *F*-to-exclude probability was 0.1. Significance levels are two-tailed.

## 3. Results

Table 1 presents mean scores for the WAIS-III, WCS, and TMT.  Table 2 presents correlations of TMT and WCS measures with WAIS-III IQs and indexes. As can be seen in Table 2, response times on Trails A and on Trails B correlated very significantly (all *p*'s < 0.001) with all WAIS-III summary measures. When controlling for response speed, as measured by Trails A, faster Trails B performance remained highly correlated (all *p*'s < 0.01) with higher WAIS-III full-scale (partial *r* = −0.424, *p* < 0.001), verbal (partial *r* = −0.315, *p* < 0.002), and performance (partial *r* = −0.449, *p* < 0.001) IQs, as well as with higher WAIS-III index scores for verbal comprehension (partial *r* = −0.279, *p* = 0.008), perceptual organization (*r* = −0.315, *p* = 0.003) working memory (*r* = −0.380, *p* < 0.001), and processing speed (partial *r* = −0.411, *p* < 0.001). By contrast, when controlling for attentional control, as measured by Trails B response time, none of these partial correlations remained significant between Trails A and WAIS-III summary measures.

As shown in Table 2, WCS categories completed correlated positively with WAIS-III full-scale (*r* = 0.330, *p* = 0.003), verbal (*r* = 0.299, *p* = 0.006), and performance (*r* = 0.250, *p* = 0.023) IQs. Similarly, fewer nonperseverative errors correlated with higher full-scale (*r* = −0.337, *p* = 0.002), verbal (*r* = −0.303, *p* = 0.005), and performance (*r* = −0.250, *p* = 0.025) IQs. Lower rates of WCS perseverative errors also correlated significantly with higher scores for WAIS-III full-scale (*r* = −0.464, *p* < 0.001), verbal (*r* = −0.445, *p* < 0.001), and performance (*r* = −0.314, *p* = 0.004) IQs as well as with WAIS-III index measures of verbal comprehension (*r* = −0.342, *p* = 0.002), perceptual organization (*r* = −0.240, *p* = 0.029), and working memory (*r* = −0.316, *p* = 0.004). Trails B response time correlated very strongly with WCS indices of perseverative (*r* = −0.372, *p* < 0.001) and nonperseverative (*r* = −0.243, *p* = 0.004) errors as well as with categories completed (*r* = −0.332, *p* < 0.001). By contrast, Trails A response time did not correlate with any of the WCS performance measures (see Table 2).

We next used hierarchical regression to examine the unique contributions of Trails B response time and WCS indexes of perseverative errors and nonperseverative errors to full-scale IQ scores. Trails B response time produced a highly significant *R* square change of 0.280 (*F* = 29.54, df = 1, 76, *p* < 0.001) as did WCS perseverative errors with a *R* square change of 0.081, (*F* = 9.55, df = 1, 75, *p* = 0.003). By contrast, WCS nonperseverative errors did not account for a significant source (*p* > 0.35) of specific variance in intelligence. Follow-up comparisons regressed Trails B response time and WCS perseverative errors on full-scale IQ scores. Results indicated that Trail B response times uniquely accounted for 15.13% to 19.18% [partial correlation = −0.438, part correlation = −0.389] of the variance in full-scale IQ. By comparison, WCS perseverative errors uniquely accounted for 8.12% to 11.29% [partial correlation = −0.336, part correlation = −0.285] of the variance in full-scale IQ. Thus, these results provided support for the hypothesis that higher levels of intellectual abilities may depend greatly on executive attentional control functions related to response inhibition and shifting mental sets, as measured by Trails B response time and WCS perseverative errors.

Table 3 presents relative volumes for the orbital frontal subregions for 25 of the healthy control participants.  Table 4 presents correlations of OFC gray matter volumes with WAIS-III, TMT, and WCS scores in participants who had undergone MRI studies. For the measures of attentional control, faster performance on Trails B correlated very significantly (*p*'s < 0.01) with greater gray matter volume for left (*r* = −0.577, *p* = 0.006) total OFC as well as for left (*r* = −0.608, *p* = 0.003) middle orbital gyrus. Likewise, fewer perseverative errors correlated with greater gray matter volumes in right OFC (*r* = −0.466, *p* = 0.029) as well as in right middle orbital gyrus (*r* = −0.450, *p* = 0.036), but these significant correlations did not survive correction for multiple comparisons across OFC subregions. For the WAIS-III summary measures, greater left OFC gray matter total volume correlated very significantly with verbal comprehension (*r* = 0.586, *p* = 0.003), as did greater right OFC gray matter total volume correlate with higher scores for full-scale (*r* = 0.615, *p* = 0.002), verbal (*r* = 0.585, *p* = 0.003), and performance (*r* = 0.591, *p* = 0.003) IQs, and for verbal comprehension (*r* = 0.632, *p* = 0.001) and perceptual organization (*r* = 0.594, *p* = 0.003) indexes. Greater right middle orbital gyrus gray matter volume correlate with full-scale (*r* = 0.610, *p* = 0.002), verbal (*r* = 0.549, *p* = 0.007), and performance (*r* = 0.620, *p* = 0.002) IQs as well as with verbal comprehension (*r* = 0.561, *p* = 0.002) and perceptual organization (*r* = 0.605, *p* = 0.002).

**Figure 1:**
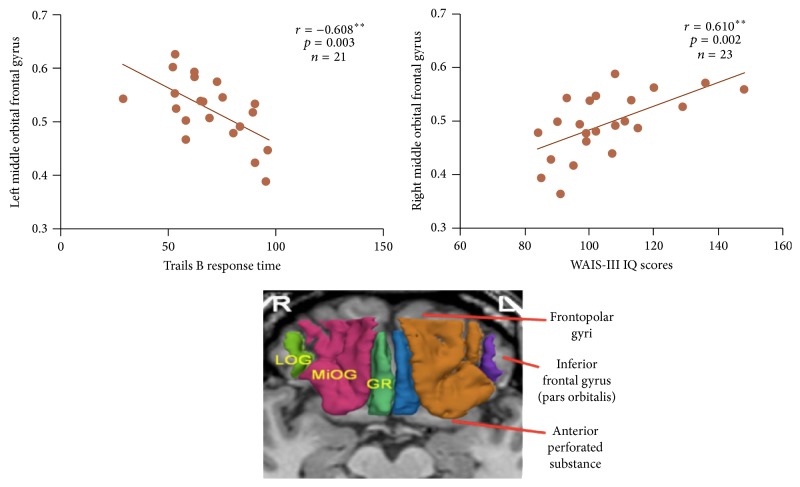
Orbital frontal cortex subregions of the lateral orbital gyrus (LOG), middle orbital gyrus (MiOG), and gyrus rectus (GR) along with scatter plots of right MiOG with WAIS-III full-scale IQ and left MiOG with Trails B response ^*∗∗*^ *p* < .01.

Figure 1 presents the scatter plots depicting the correlation of left middle orbital gray matter volume and Trails B response time (*r* = −0.608, *p* < 0.003) and the correlation of right middle orbital gray matter volume and full-scale IQ (*r* = 0.610, *p* < 0.002). As a test of the attentional control hypothesis of intelligence, we used hierarchical regression to examine the unique contributions of right middle orbital gray matter volume and Trails B response time to IQ scores. The results showed that each independent variable made a significant and unique contribution to full-scale IQ, producing highly significant *R* square changes of 0.347 for right middle orbital frontal gyrus gray matter volume (*F* = 10.08, df = 1, 19, *p* = 0.005) and 0.255 (*F* = 11.54, df = 1, 18, *p* = 0.003) for Trails B response time. Full-scale IQ and right middle orbital gyrus volume had semipartial and partial correlation values of 0.273 and 0.397, respectively, in comparison to −0.505 and −0.625 for full-scale IQ and Trails B response time. These values indicated that right middle orbital gyrus volume uniquely accounted for 7.45% to 15.76% of the variance in IQ in comparison to 25.50% to 39.06% uniquely explained by Trails B response time, and together, Trails B response time and middle orbital gyrus gray matter volume accounted for approximately 32.95% to 54.82% of the variance in IQ scores. Figure 1 depicts scatter plots of right middle orbital gyrus gray matter volume with WAIS-III full-scale IQ and with Trails B response time.

## 4. Discussion

The current study combined neuropsychological and structural brain imaging measures to examine componential neural and informational processes underlying individual differences in intellectual abilities. The results suggested that normal variation in higher-order cognition, as measured by full-scale IQ of the WAIS-III, may be influenced to a significant degree by individual differences in basic attention-control capacities related to response inhibition and shifting mental set. By contrast, the strong relationship of attentional control capacity and IQ occurred independently of any differences in processing speed. In addition, data from structural imaging of three subregions of the OFC provided evidence of a rather strong and specific relationship of increased bilateral gray matter volumes of the middle orbital gyrus with both greater attentional control capacity and intellectual abilities. Taken together, these findings are consistent with emerging models that posit that prefrontal contributions to cognitive intelligence are mediated in part by attention-control capacity, [31, 32] which, in these current data was, supported, structurally, by gray matter volume of the middle orbital gyrus.

The current findings may thus add to the growing body of research aimed towards elucidating the mental and neural architecture of human psychometric intelligence (e.g., [13, 33, 34]). In particular, the current results may be viewed complementary to other neuroscience-based models that have outlined a “neural efficiency theory” of intelligence (e.g., [35]). In these models, information and neuronal processing speed is thought to reflect the efficiency of key neuropsychological mechanisms supporting higher intelligence (e.g., [1, 14]). Consistent with this line of reasoning, recent brain imaging studies have shown that individual variation in intelligence is heavily influenced by the structural integrity of corresponding white matter connections and association tracts that allow for efficient neural transmission across proximate and distal brain areas [14, 36], particularly frontal and parietal regions [6].

The current study, by contrast, focused on the relationship of OFC gray matter volumes with attention-control capacity and intelligence. We employed hierarchical regression analyses to examine the three-way relationship among middle orbital gyrus gray matter volume, attentional control, and intelligence. These results indicated that right middle orbital gyrus gray matter volume and attentional control each accounted for a unique and significant source of variance in IQ scores. That is, attentional control capacity, as measured by Trails B response speed, explained approximately 25.50% to 39.06% of the IQ variance with an additional 7.45% to 15.76% of individual differences in intelligence explained by right middle orbital gyrus gray matter volume.

How might greater MRI prefrontal gray matter volumes influence both attention-control capacity and intelligence? To the extent that the MRI signal captures key cellular properties of the brain, increased prefrontal gray matter volume might reflect the density and number of neuronal bodies and dendritic expansions available to support mental computation and abstraction, which in turn may lead to improved cognitive functioning [37]. In fact, numerous structural brain imaging studies have found strong positive correlation between prefrontal gray matter volume and intelligence (e.g., [37]). In addition, findings from functional brain imaging studies have suggested a critical role of prefrontal cortex-mediated working memory in intelligence [34]. Here the current study extended these findings by demonstrating a particular region of the prefrontal cortex, the right middle orbital gyrus, as strongly linked to both attentional control and intelligence, perhaps providing important neurobiological substrata for attention-control capacity necessary for instrumental learning [38].

In summary, the current findings pointed to a specific and strong role of attention-control capacity in intelligence, which may be mediated in large part by OFC structures, particularly the middle orbital gyrus. However, the current study is limited by its correlational design and its region-of-interest focus on OFC structures, which did not allow for examination of intelligence in relation to whole-brain network properties. Future experimentally based studies using both structural and functional imaging may help to elucidate further the distributed and dynamic nature of the neural and informational mechanisms that give rise to intelligence.

## Figures and Tables

**Table 1:** Neuropsychological scores for research participants.

Demographic information	
Age	40.80 ± 9.08
Education	14.78 ± 2.07
SES	2.30 ± 1.01
WAIS-III IQ	
Full scale	109.20 ± 13.70
Verbal	109.83 ± 13.32
Performance	106.72 ± 14.50
WAIS-III index	
Verbal comprehension	108.04 ± 12.82
Perceptual organization	107.75 ± 14.83
Working memory	108.83 ± 14.66
Processing speed	105.66 ± 15.04
Trail Making Test (seconds)	
Trails A	31.14 ± 11.87
Trails B	67.81 ± 27.07
Wisconsin Card Sort	
Categories completed	5.32 ± 1.48
Perseverative errors	12.36 ± 11.81
Nonperseverative errors	12.14 ± 11.12

*Note.* Values are means plus or minus standard deviations. SES = socioeconomic status; WAIS-III = Wechsler Adult Intelligence Scale-Third Edition, WMS-III.

**Table 2:** Correlations of WAIS-III summary measures with Trail Making and Wisconsin Card Sort.

	Trails Making		Wisconsin Card Sort		
	Trails A	Trails B	CC	PE	NPE
WAIS-III IQ					
Full scale	−0.355∗∗∗	−0.558∗∗∗	0.330∗∗	−0.464∗∗∗	−0.0337∗∗
Verbal	−0.363∗∗∗	−0.408∗∗∗	0.299∗	−0.445∗∗∗	−0.303∗∗
Performance	−0.383∗∗	−0.572∗∗	0.250∗	−0.314∗∗	−0.250∗
WAIS-III index					
Verbal Comprehension	−0.350∗∗∗	−0.372∗∗∗	0.153	−0.342∗∗	−0.16
Perceptual organization	−0.408∗∗∗	−0.421∗∗∗	0.149	−0.240∗	−0.152
Working memory	−0.431∗∗∗	−0.422∗∗∗	0.185	−0.316∗∗	−0.177
Processing speed	−0.515∗∗∗	−0.471∗∗∗	0.072	−0.207	−0.87

∗*p* < 0.05, ∗∗*p* < 0.01, ∗∗∗*p* < 0.001.

*Note.* CC = categories completed; PE = perseverative errors; NPE = nonperseverative errors.

**Table 3:** Relative volumes for orbital frontal cortex subregions.

OFC subregion	Mean (±standard deviation)
Gyrus rectus	
Left	0.161 (±0.026)
Right	0.174 (±0.026)
Middle orbital Gyrus	
Left	0.520 (±0.056)
Right	0.495 (±0.056)
Lateral orbital Gyrus	
Left	0.050 (±0.014)
Right	0.054 (±0.012)

*Note*. Values are means plus or minus standard deviations.

Relative volume = [Absolute Volume (cm^3^)/Intracranial Contents Volume (cm^3^)] × 100 (%).

**Table 4:** Pearson correlations of orbital frontal cortex volumes and neuropsychological measures.

Measures	Left				Right			
	GR	MiOG	LOG	OFC_L	GR	MiOG	LOG	OFC_R
WAIS-III IQ								
Full scale	0.057	0.481∗	0.006	0.435∗	0.251	0.610∗∗	0.11	0.615∗∗
Verbal	0.036	0.511∗	0.042	0.461∗	0.307	0.549∗∗	0.113	0.585∗∗
Performance	0.054	0.420∗	−0.018	0.377	0.157	0.62∗∗	0.113	0.591∗∗
WAIS-III index								
Verbal comprehension	0.099	0.622∗∗	0.073	0.586∗∗	0.396	0.561∗∗	0.148	0.632∗∗
Perceptual organization	0.075	0.345	−0.082	0.307	0.218	0.605∗∗	0.081	0.594∗∗
Working memory	0.094	0.166	0.090	0.196	0.203	0.348	0.076	0.375
Processing speed	0.145	0.296	0.116	0.332	0.212	0.372	0.141	0.409
Trail Making Test (in seconds)								
Trails A	0.268	−0.370	0.509∗	−0.146	0.111	−0.307	0.139	−0.209
Trails B	−0.083	−0.608∗∗	−0.072	−0.577∗∗	−0.169	−0.481∗	−0.222	−0.521∗
Wisconsin Card Sort								
Categories completed	−0.066	0.181	−0.145	0.111	−0.128	0.164	0.184	0.124
Perseverative errors	−0.25	−0.399	0.193	−0.406	−0.162	−0.450∗	−0.046	−0.466∗
Nonperseverative errors	0.036	−0.23	0.162	−0.163	0.084	−0.134	−0.176	−0.112

∗∗Correlation is significant at the 0.01 level (2-tailed).

∗Correlation is significant at the 0.05 level (2-tailed).

*Note.* GR = gyrus rectus, MiOG = m middle orbital gyrus, LOG = lateral orbital gyrus, and OFC = orbitofrontal cortex.
